# Immune recognition of salivary proteins from the cattle tick *Rhipicephalus microplus* differs according to the genotype of the bovine host

**DOI:** 10.1186/s13071-017-2077-9

**Published:** 2017-03-14

**Authors:** Gustavo Rocha Garcia, Sandra Regina Maruyama, Kristina T. Nelson, José Marcos Chaves Ribeiro, Luiz Gustavo Gardinassi, Antonio Augusto Mendes Maia, Beatriz Rossetti Ferreira, Frans N. J. Kooyman, Isabel K. F. de Miranda Santos

**Affiliations:** 1 Department of Biochemistry and Immunology, Ribeirão Preto School of Medicine, University of São Paulo, Ribeirão Preto, São Paulo Brazil; 2 Center for the Study of Biological Complexity, Virginia Commonwealth University, Richmond, VA USA; 3 Laboratory of Malaria and Vector Research, National Institute of Allergy and Infectious Diseases, National Institutes of Health, Rockville, MD USA; 4 Department of Basic Sciences, School of Animal Science and Food Technology, University of São Paulo, Pirassununga, São Paulo Brazil; 5 Department of Maternal-Child Nursing and Public Health, Ribeirão Preto School of Nursing, USP, Ribeirão Preto, SP Brazil; 6 Department of Infectious Diseases and Immunology, Faculty of Veterinary Medicine, Utrecht University, Utrecht, The Netherlands

**Keywords:** Immunoglobulins, Proteome, Immunoproteome, Antibody response, *Rhipicephalus microplus*, Tick saliva, *Bos taurus*, *Bos indicus*

## Abstract

**Background:**

Males of the cattle tick *Rhipicephalus microplus* produce salivary immunoglobulin-binding proteins and allotypic variations in IgG are associated with tick loads in bovines. These findings indicate that antibody responses may be essential to control tick infestations. Infestation loads with cattle ticks are heritable: some breeds carry high loads of reproductively successful ticks, in others, few ticks feed and they reproduce inefficiently. Different patterns of humoral immunity against tick salivary proteins may explain these phenotypes.

**Methods:**

We describe the profiles of humoral responses against tick salivary proteins elicited during repeated artificial infestations of bovines of a tick-resistant (Nelore) and a tick-susceptible (Holstein) breed. We measured serum levels of total IgG1, IgG2 and IgE immunoglobulins and of IgG1 and IgG2 antibodies specific for tick salivary proteins. With liquid chromatography followed by mass spectrometry we identified tick salivary proteins that were differentially recognized by serum antibodies from tick-resistant and tick-susceptible bovines in immunoblots of tick salivary proteins separated by two-dimensional electrophoresis.

**Results:**

Baseline levels of total IgG1 and IgG2 were significantly higher in tick-susceptible Holsteins compared with resistant Nelores. Significant increases in levels of total IgG1, but not of IgG2 accompanied successive infestations in both breeds. Resistant Nelores presented with significantly higher levels of salivary-specific antibodies before and at the first challenge with tick larvae; however, by the third challenge, tick-susceptible Holsteins presented with significantly higher levels of IgG1 and IgG2 tick salivary protein-specific antibodies. Importantly, sera from tick-resistant Nelores reacted with 39 tick salivary proteins in immunoblots of salivary proteins separated in two dimensions by electrophoresis *versus* only 21 spots reacting with sera from tick-susceptible Holsteins.

**Conclusions:**

Levels of tick saliva-specific antibodies were not directly correlated with infestation phenotypes. However, in spite of receiving apparently lower amounts of tick saliva, tick-resistant bovines recognized more tick salivary proteins. These reactive salivary proteins are putatively involved in several functions of parasitism and blood-feeding. Our results indicate that neutralization by host antibodies of tick salivary proteins involved in parasitism is essential to control tick infestations.

**Electronic supplementary material:**

The online version of this article (doi:10.1186/s13071-017-2077-9) contains supplementary material, which is available to authorized users.

## Background

*Rhipicephalus microplus*, the cattle tick, threatens animal health and cattle production worldwide [1]. The direct and indirect effects of infestations by *R. microplus* cause losses in the order of billions of dollars annually [2, 3]. In Brazil, home to the largest commercial herd of cattle, the losses exceed 3.24 billion dollars a year [4]. Despite these losses, a reliable and sustainable method of tick control is not available. The available anti-tick vaccines offer partial and transient protection and the chemical agents result in environmental contamination, residues in food products and promote acaricide-resistant ticks [5, 6].

Bovine hosts present contrasting and heritable phenotypes for tick infestations [7, 8]. Breeds of *Bos indicus* (indicine) cattle are more resistant to *R. microplus* than those of *B. taurus* (taurine) cattle, possibly because the former were domesticated in Asia [9, 10], which is also believed to be the place of origin of the parasite [11]. Even after they undergo repeated infestations, taurine breeds still remain susceptible to levels of tick loads that are unacceptable in terms of animal productivity and health [7, 8, 12–16]. Knowledge about the mechanisms involved in the hosts’ resistance to ticks will point to the path of new strategies of tick control.

Tick saliva is responsible for the success of parasite attachment, blood-feeding and transmission of pathogens to hosts [17, 18]. It is a complex xenobiotic substance that is composed of soluble proteins presenting an array of different functions. In the model for studying tick-host interactions employed in the present study, saliva is constantly inoculated into the host for three weeks and, by accounting for the saliva produced by each tick throughout the infestation [19], highly infested hosts can receive approximately 200 ml of saliva and protein to the level of milligrams. Given the importance of antibodies in neutralizing venoms and salivary mediators of parasitism, we and others [20–22] have made several observations on antibody responses made by bovine hosts against tick antigens. While hosts indeed produce antibodies against tick salivary proteins, a tenet of immunology is that soluble antigens administered in large quantities without aggregation or adjuvants are not immunogenic [23, 24], which is the case at the host-tick interface. Therefore, neutralization of tick salivary mediators of parasitism by host antibodies might be compromised in tick-susceptible taurine hosts.

We have previously described the antibody responses to tick saliva in bovine hosts that were managed in pastures naturally infested with high or low numbers of ticks [25]. We reported that, in spite of less ticks feeding on tick-resistant bovines (Nelore breed, *B. indicus*), these hosts presented with significantly higher levels of tick saliva-specific IgG1 and IgG2 antibodies than tick-susceptible bovines (Holstein breed, *B. taurus*) when the latter hosts are presenting with heavy tick loads. Furthermore, in another tick-susceptible breed (Aberdeen) only levels of IgG1, but not IgG2 saliva-specific antibodies were positively correlated with tick loads acquired in naturally infested pastures. Cruz and colleagues [26] obtained similar results when they examined total IgG saliva-specific antibody responses after artificial infestations in the tick-susceptible Hereford breed. A study by Piper and colleagues [27] examined differences in antibody responses mounted by tick-resistant Brahmans and tick-susceptible Holsteins artificially infested with ticks and found that IgG1, but not IgG2 tick-specific antibodies were significantly higher in susceptible hosts than in resistant hosts.

Clearly more information is needed about the development of the antibody response to tick saliva. The present study sought to generate more information on antibody responses to ticks. For this, we examined the antibody responses to different sets of tick salivary antigens in the model of contrasting phenotypes of infestations between tick-resistant (Nelore breed, *B. indicus*) and tick-susceptible (Holstein breed, *B. taurus*) bovines undergoing artificial infestations with larvae of *R. microplus* ticks. Serum samples were collected in hosts kept free of ticks until the first infestation and during sequential stages of the parasite’s life-cycle for three successive infestations. Levels of total IgG1, IgG2, IgE and of IgG1 and IgG2 antibodies specific for saliva and for extracts of female salivary glands (FSG) were measured through enzyme-linked immunosorbent assays (ELISA). In addition, the recognition of antigens from female tick saliva and extracts of unfed larvae (UFL), of male salivary glands (MSG) and of FSG by bovine IgG antibodies was evaluated by immunoblotting tick proteins separated in one dimension; the identity of reactive antigens from female tick saliva was evaluated by immunoblotting tick proteins separated in two dimensions followed by spot picking and sequencing proteins of gels prepared in parallel. We show that bovines presenting contrasting tick loads, i.e. animals of Nelore and Holstein breeds, present significant differences in their levels of total IgG1, IgG2 and IgE and in their antibody responses against tick antigens along successive infestations. Furthermore, in spite of a much lower exposure to tick antigens, resistant Nelore hosts recognize a larger set of tick salivary antigens.

## Methods

### Hosts, phenotypes of infestations and experimental design for collection of sera

Animals entered the experiment at six months of age before contact with *R. microplus* ticks. These were non-related, six-months old calves, four of the Nelore breed (genetically tick-resistant, *Bos taurus indicus*), and four of the Holstein breed (genetically tick-susceptible, *Bos taurus taurus*). They were kept stabled at the University of São Paulo’s farm, located in Pirassununga, São Paulo State, Brazil. The calves were maintained free of ticks from birth with the following measures: the pregnant mothers were strategically treated with acaricides and maintained in a clean pasture, the newborn calves were housed in sand hutches during the weaning period and were submitted to strategic acaricide treatments. At the beginning of the experiment sera were collected from calves after which they were infested artificially with approximately 10,000 15-day-old unfed larvae of *R. microplus* from our colony [28, 29] maintained on Holstein oxen and originated from ticks collected at the University farm at Pirassununga. The phenotypes for resistance and susceptibility to ticks of the two breeds were confirmed by counting female ticks larger than 4 mm on the left side of each animal on the 21st day after release of larvae for the first infestation and these results and the procedure performed with the experimental animals used in the present study has been previously described [30].

For studies of antibody responses against saliva and FSG, sera were collected from calves before contact with ticks and when they were infested with ticks at the following stages of their life-cycle: two days after larvae were released and 7 and 15 days after infestation, when larvae had molted to nymphs and adults, respectively; three successive infestations were made with an interval of two months between the first and second and an interval of three months between the second and third; seven months lapsed between the beginning of the first infestation and the end of the last. Serum samples were obtained from peripheral blood collected in Vaccutainer® tubes without anticoagulants followed by centrifugation and inactivation at 56 °C for 30 min. All samples were aliquoted and stored at −20 °C until use. Characteristics of sera evaluated in this study are described in Additional file 1: Table S1.

### Ticks

In order to obtain saliva, 400 semi-engorged female ticks were taken directly from susceptible hosts (Holstein breed), washed, dried and injected in the haemocoele with a solution of dopamine (Revivan®, Zambon, São Paulo, Brazil). Saliva was collected in protease inhibitors according to manufacturer’s recommendations (Complete, Mini EDTA-free, Roche, Basel, Switzerland) and frozen at -20 °C. FSG and MSG were collected from ticks fed on susceptible (hereafter designated FSGS or MSGS) and resistant hosts (FSGR or MSGR) under a dissecting microscope and with sterile dissection tools and the glands were put in water with protease inhibitors. The samples were sonicated in order to obtain the extract. The extracts of UFL were obtained from 1 mg of egg masses from female ticks fed on resistant or susceptible hosts (hereafter designated UFLS and UFLR). The larvae were put in water with protease inhibitors according to the manufacturer’s recommendations (Complete, Mini EDTA-free, Roche, Basel, Switzerland), followed by pulverization using a pestle and liquid nitrogen in order to obtain the extract. Proteins concentrations were measured by the Coomassie assay, according to the manufacturer’s instructions (Pierce, Rockford, IL, USA).

### Measurement of total IgG1, IgG2 and IgE and detection and measurement of tick anti-salivary protein IgG1 and IgG2 antibodies

#### Enzyme-linked immunosorbent assays (ELISA)

Levels of total IgG1 and IgG2 from cattle were measured in sera described in Additional file 1: Table S1 using a Bovine IgG1 and IgG2 ELISA quantification Kit (Bethyl Labs, Montgomery, TX, USA) according to the manufacturer’s instructions. The measurement of levels of total IgE was performed according to the ELISA protocol developed by Kooyman and colleagues [31]. Briefly, monoclonal anti-sheep IgE diluted to 1:100 was used as capture antibody and test sera were diluted 1:5 and incubated on the plates, followed by incubation with a second, polyclonal rabbit anti-bovine IgE antibody solution diluted 1:250. Afterwards, goat anti-rabbit IgG antibody conjugated with alkaline phosphatase (Dako, Glostrup, Denmark) was used at a 1:1000 dilution and a color was developed with p-nitrophenyl phosphatase chromogenic solution (Sigma Chemical Company, St. Louis, USA) after incubation during 30 min at room temperature and overnight at 4 °C. The measurement of levels of saliva and FSG extract-specific IgG1 and IgG2 antibodies were performed according to the ELISA protocol developed by Kashino et al. [25]. Results are expressed as the values of absorbance at 450 nm minus the absorbance of duplicate blank samples included in each plate. The end-point titers displayed were determined to be the last of serial twofold dilutions presenting a significant difference in the optical density at 450 nm when compared to that seen in the corresponding dilution of sera from a different experimental group.

#### Immunoblotting of tick antigens

##### Immunoblotting of tick salivary proteins separated in one dimension

FSG extracts (14 μg), MSG extracts (8 μg) and UFL extracts (24 μg) were separated using 12.5% SDS polyacrylamide gels on a vertical unit (BioRad, Hercules, California, USA), with the initial amperage at 15 mA/gel for 15 min followed by 30 mA/gel to total separation. After resolving in one dimension, the extracts were transferred from polycrylamide gels to Hybond ECL nitrocellulose sheets (GE Healthcare BioSciences Corporation, Piscataway, USA) using TE 70 semi-dry transfer unit (GE Healthcare BioSciences Corporation, Piscataway, USA) and pre-stained protein standards (BioRad, Hercules, California, USA) were used to monitor blot transfer. These membranes were incubated with pooled sera using dilutions at 1:75 for 4 h at 37 °C. After washing, the membranes received the peroxidase conjugated Protein G (Thermo Scientific Pierce, Waltham, Massachusetts, USA) diluted at 1:2500 and ECL Western Blotting Substrate (Thermo Scientific Pierce, Waltham, Massachusetts, USA) to detect the antigens reactive with sera from different timepoints through chemiluminescence in an ImageQuant 350 detection system (GE Healthcare BioSciences Corporation, Piscataway, USA). Proteins in samples similar to those described above were separated under the same conditions and stained using a kit of PlusOne Coomassie Tablets (GE Healthcare BioSciences Corporation, Piscataway, USA) in order to verify their protein profiles.

##### Immunoblotting of tick salivary proteins separated in two dimensions and Spot Picking

The two-dimensional electrophoreses were made in 13 cm strips of immobilized gradient (IPG) at pH 3-10NL (GE Healthcare BioSciences Corporation, Piscataway, USA). The amount of protein sample used per strip was 200 μg (except to gel used for spot picking that was 400 μg) and six gels were run in parallel for spot picking for identification of proteins and for immunoblotting to identify the salivary components that react with sera from tick-naïve and tick infested susceptible and resistant hosts. The saliva was treated with 100% trichloroacetic acid to eliminate residual dopamine at a final concentration of 10% for 1 h at 4 °C. The proteins were then centrifuged at 14,000 rpm at 4 °C for 15 min, the supernatant discarded and the pellet was washed three times with acetone (500 μl of acetone for each 1 ml of saliva) and centrifuged at 10,000 rpm for 10 min. After washing with acetone, the pellet was re-suspended in rehydration solution (7 M of urea, 2 M tiourea, 4% CHAPS, 20 mM DTT). The strips were rehydrated for 10–14 h. Thereafter, electrophoresis in strips was performed on an IPGPhor equipment (GE Healthcare Bio-sciences Corp, Piscataway, USA) according to the manufacturer’s recommendations for IPG strips of 13 cm (i.e. 1 step at 500 V during 1 h, followed by gradient of 1,000 V and 8,000 V for 1 h and 2 h 30 min, respectively; and a finishing step of 8,000 V during 1 h, totaling 5 h 30 min or until 16–20 kVh are accumulated). The strips were balanced in a solution containing SDS and urea (50 mM Tris, 6 M urea, 2% SDS, 30% glycerol, pH 8.8) and DTT (100 mg/10 ml) for 15 min under mild shaking, and then iodacetamide (250 mg/10 ml) for another 15 min under mild shaking. The second dimension of resolution was run in 12.5% SDS polyacrylamide gels on a SE600 (GE Healthcare Bio-sciences Corp, Piscataway, USA), with the initial amperage at 15 mA/gel for 15 min followed by 30 mA/gel until total separation. After running, the 2D gels were stained with silver nitrate or electrotransferred onto PVDF membrane at 10 V, 400 mA for 25 min in transfer buffer (48 mM Tris, 39 mM Glycine, 20% methanol and 0.375% SDS) using ECL semi-dry transfer unit (GE Healthcare BioSciences Corporation, Piscataway, USA). The membranes were blocked for 14 h with 5% skimmed milk diluted in TBS-T (50 mM Tris, 0.1 M NaCl, 0.05% Tween, pH 7.2). They were then washed twice for 5 min with TBS-T and then incubated with pooled sera diluted 1:100 in TBS-T 0.01% Azide and 2.5% bovine serum albumin with shaking for 14 h at 4 °C followed by washing 3 times for 5 min with TBS-T. To verify the antibody-antigen reaction G protein conjugated with peroxidase (Invitrogen Corporation, Carlsbad, CA) was employed diluted 1:2000 and incubated with the membranes under agitation for 1 hat 37 ° C, followed by 3 washes for 5 min with TBS-T and incubation with ECL Western Blotting Substrate (Thermo Scientific Pierce, Waltham, Massachusetts, USA) and the visualization of the spots was shown using the Multifunctional Imaging System (GE Healthcare Bio-sciences Corp, Piscataway, USA). The gel model was used to collect the spots of interest based on the results of western blots. The spots were collected using individual sterile pipette tips into tubes containing sterile water and stored until sequencing.

### Sequencing of tick salivary proteins by liquid chromatography and mass spectrometry (LC-MS)

The spots previously selected and collected were transferred to a new tube, washed and destained with 500 μl of a 50% methanol solution for 14 h. The spots were dehydrated in 200 μl of acetonitrile and rehydrated in 30 μl of 10 mM DTT in 0.1 M ammonium bicarbonate and reduced for 30 min at RT. The DTT solution was removed and the samples were alkylated in 30 μl of 50 mM iodoacetamide in 0.1 M ammonium bicarbonate at RT for 30 min. The solution was removed and the spots were dehydrated in 100 μl of acetonitrile. Acetonitrile was removed and the spots were rehydrated in 100 μl of 0.1 M ammonium bicarbonate. Subsequently, the spots were dehydrated again in 100 μl of acetonitrile and this solution was then removed and the spots were completely dried by vacuum centrifugation. Spots were then rehydrated in 20 ng/μl of trypsin in 50 mM ammonium bicarbonate for 10 min on ice, followed by digestion 14 h at 37 °C. Afterwards, any excess trypsin solution was removed by pipetting and 20 μl of 50 mM ammonium bicarbonate were added. The peptide fragments were placed in two 30 μl aliquots of 50% acetonitrile in 5% formic acid. These extracts were combined and evaporated for sequencing with mass analysis.

For LC-MS, we employed a hybrid LTQ-Orbitrap mass spectrometer Thermo Electron system with an ion source “nanospray” connected to a reverse phase capillary column C18 Waters NanoAcquity (Thermo Scientific Pierce, Waltham, Massachusetts, USA). Approximately 2–5 μg of peptides were injected into these columns. Peptides that were not attached to the SCX column (C18) were then eluted by an acetonitrile gradient in 0.1 M formic acid with a flow rate of 0.4 μl/min for 1 h. After this continuous flow fractionation, eight fractions of ion exchange were eluted on a C18 column, using aliquots of increasing concentration of ammonium acetate. Each fraction was analyzed by an acetonitrile gradient in 0.1 M formic acid with a flow rate of 0.4 μl/min. for 1 h. The source of nanospray ion was operated at 3.5 kV.

The output data from each sample were analyzed in search database using Sequest search algorithm. Our sialotranscriptome of *R. microplus* served as a database for these searches (BioProject ID PRJNA329522). The peptides and proteins identified in the samples were inputed into Scaffold format file. The reliability of peptides identification was evaluated using the following criteria analysis in Scaffold software: (i) minimum of two unique peptides for each protein identified; (ii) 99% minimum probability of protein identification; and (iii) 95% minimum probability peptide identification. The bioproject that contained the sequences obtained from sialotranscriptome of *R. microplus* informed above served as tick database for identification of peptides isolated from immunoproteomes and is deposited at GenBank (NCBI): *Rhipicephalus microplus*: BioProject ID 329522.

### Statistical analysis

Statistical analyses were performed using the Student’s *t* test and the one-way analysis of variance (ANOVA) to evaluate significance among groups and the influence of two different variables on one continuous variable, respectively. *P*-value < 0.05 was used to establish the level of significance. Tukey’s multiple comparisons test (*post-hoc* analysis) was used to find means that are significantly different from each other. GraphPad Prism 6 version 6.01 (San Diego, CA, USA) was used to perform the statistical tests.

## Results

The cattle tick *R. microplus* is a one host (monoxene) tick and spends the entire parasitic stage of its life-cycle, approximately three weeks, on the same host. This period is sufficient for the host to mount primary and secondary antibody responses against those tick salivary components that are produced at the beginning or over the entire parasitic stage. Highly infested hosts receive large amounts of tick saliva, which contains many immunosupressants, including immunoglobulin-binding proteins, which are abundantly transcribed in salivary glands of the feeding male tick [32], coinciding with the appearance of the host’s acquired immune response. The genetic composition of hosts affects the nature of the antibody response, including levels of total immunoglobulins [33–37], the IgG subclasses recruited, and antibody specificities [33, 38]. On the other hand, ticks ingest large amounts of host immunoglobulins presenting antibody activity [39]. In view of these data we examined several aspects of the humoral response in bovines of an indicine and of a taurine breed known to be genetically resistant or susceptible, respectively, to the cattle tick, *R. microplus*. Ticks loads in the Nelore and Holstein hosts examined in the present study were assessed as previously described [30], confirming the significant differences between these breeds.

### The effect of breed and of tick infestations on levels of total IgG1, IgG2 and IgE immunoglobulins in different breeds of bovines

**Fig. 1:**
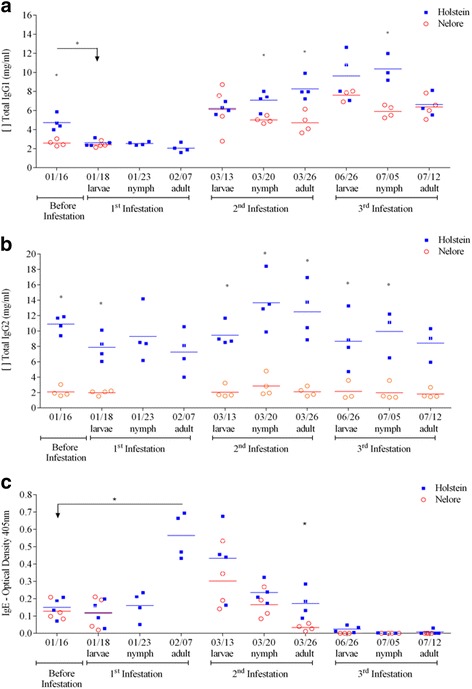
Amounts of total IgG1, IgG2 and IgE immunoglobulins differ between tick-resistant and tick-susceptible breeds of cattle. Amounts of total IgG1 (**a**), IgG2 (**b**) and IgE (**c**) immunoglobulins were measured in animals of a tick-susceptible (Holstein, *blue* squares) and a tick-resistant (Nelore, *red open circles*) breed of cattle before (baseline levels) and during three successive artificial infestations with ticks, when parasites were at the larval, nymphal and adult stages of their life-cycle. Dilutions of sera employed in indirect ELISAs were 1:100 and 1:5 for total IgG1 and IgG2, and for total IgE, respectively. Asterisks indicate the levels of significance between amounts of total IgG1, IgG2 and IgE immunoglobulins from Holstein and Nelore hosts and the specific statistical results are described in the text

We first measured the serum immunoglobulins from both breeds before they were exposed to *R. microplus*. The results of ELISA show that the baseline levels (i.e. before infestations) of total IgG1 and IgG2 differ significantly between resistant and susceptible hosts (*t*_(4.878)_ = 6, *P* = 0.0028 and *t*_*(*13.43)_ = 6, *P* < 0.0001, respectively; Fig. 1a, b). Regarding levels of total IgE (Fig. 1c), no differences were seen between the two breeds.

We then examined levels of total IgG1, IgG2 and IgE in the same hosts during three successive artificial infestations with 10,000 tick larvae at the time points corresponding to the three developmental stages of the tick: larvae, nymph or adult. During the first infestation, levels of total IgG1 decreased significantly (ANOVA, *F*_(1.482, 4.447)_ = 6.386, *P* = 0.0533) in tick-susceptible Holsteins relative to baseline levels during all developmental stages of the tick (i.e. larvae, nymphs and adults; Fig. 1a) and similar differences were observed for total IgG2, but were not significant (Fig. 1b); relative to baseline levels, the levels of total IgG1 and IgG2 (Fig. 1a, b, respectively) did not change significantly in tick-resistant Nelore hosts.

During the second and third infestations the levels of total IgG1 increased in both breeds, but were significantly higher in Holsteins when compared with Nelores (*t*-test, *t*_(3.867)_ = 6, *P* = 0.0083 and *t*_(5.613)_ = 5, *P* = 0.0025, respectively; Fig. 1a). The levels of total IgG2 were, in general, significantly (first infestation: *t*-test, *t*_(6.631)_ = 6, *P* = 0.0006 (larval stage); second infestation: *t*-test, *t*_(8.785)_ = 6, *P* = 0.0001, *t*_(5.709)_ = 6, *P* = 0.0013 and *t*_(5.695)_ = 6, *P* = 0.0013; third infestation: *t*-test, *t*_(2.999)_ = 5, *P* = 0.0301, *t*_(5.018)_ = 5, *P* = 0.0040 and *t*_(5.819)_ = 5, *P* = 0.0021; larval, nymphal and adult stages, respectively) higher in Holsteins than in Nelores during the three successive infestations (Fig. 1b).

Relative to baseline levels, levels of total IgE increased significantly (ANOVA, *F*_(1.463, 4.389)_ = 13.94, *P* = 0.0144) in the tick-susceptible breed at the end of the first and beginning of the second infestations (Fig. 1c); the levels of total IgE in the resistant breed also increased during the second infestation, but the differences were not significant (Fig. 1c). Interestingly, in both breeds levels of total IgE tended to decrease during successive infestations (Fig. 1c). By the third infestation, the levels of total IgE had returned to basal levels in both breeds (Fig. 1c).

In summary, the levels of total IgG1 and IgG2 immunoglobulins are higher in the susceptible breed before and during infestations, whereas the levels of total IgE are always similar in both breeds.

### Levels of tick salivary protein-specific IgG1 and IgG2 antibodies differ in genetically tick-susceptible and tick-resistant hosts after successive infestations

**Fig. 2:**
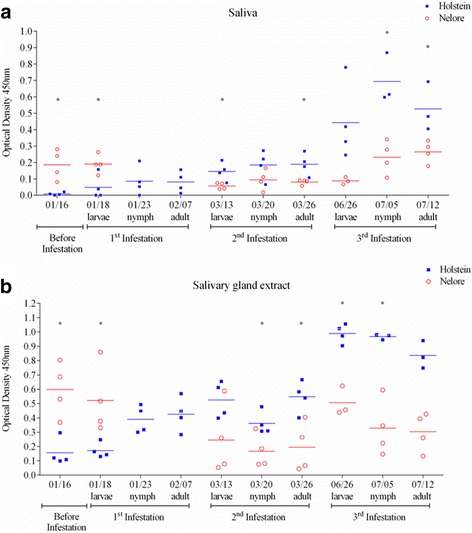
Levels of IgG1 antibodies specific for saliva and extracts of salivary glands from female ticks differ between tick-resistant and tick-susceptible breeds of cattle after successive infestations with *R. microplus*. Levels of IgG1 antibodies specific for saliva (**a**) and for salivary gland extracts from female ticks (**b**) were measured in sera from animals of a tick-susceptible (Holstein, *blue* squares) or tick-resistant (Nelore, *red open circles*) breed of cattle. Antibodies were measured before (baseline levels) and during three successive artificial infestations with ticks, when the parasites were at the larval, nymphal and adult stages of their life-cycle. A dilution of 1:100 was used for each serum. Asterisks indicate the levels of significance between amounts of specific IgG1 antibodies in Holstein and Nelore hosts and the specific statistical results are described in the text

The significant increase in levels of total IgG1 and IgG2 seen in susceptible hosts after they are infested with ticks could be due to production of IgG antibodies in response to salivary antigens that ticks salivate into their hosts. We therefore measured the levels of IgG1 and IgG2 antibodies specific for saliva from female ticks and for extracts of FSG in sera from resistant (i.e. Nelore) and susceptible (Holstein) hosts that had been kept free of ticks and were then infested artificially three times with 10,000 unfed larvae. Susceptible bovine hosts theoretically receive much larger quantities of female saliva than resistant hosts due to the significantly higher number of ticks finishing their life-cycle on them. In spite of these differences in tick loads, and despite the fact that tick-resistant bovines present with significantly lower levels of total IgG1 immunoglobulins than susceptible hosts, we verified that before exposure to ticks and at the beginning of the first infestation, levels of saliva- and FSG-specific IgG1 antibodies were significantly (*t*-test, *t*_(3.876)_ = 6, *P* = 0.0082 and *t*_(4.186)_ = 6, *P* = 0.0058, respectively) higher in tick-resistant hosts than in tick-susceptible hosts (Fig. 2a, b). Titration of sera in an end-point dilution assay confirmed these results (data not shown). However, by the third infestation, susceptible hosts produced significantly (saliva: *t*-test, *t*_(4.876)_ = 5, *P* = 0.0046 and *t*_(3.182)_ = 5, *P* = 0.0245, nymphal and adult stages, respectively; FSG: *t*-test, *t*_(7.667)_ = 5, *P* = 0.0006, *t*_(4.495)_ = 5, *P* = 0.0064 and *t*_(5.779)_ = 5, *P* = 0.0022*,* larval, nymphal and adult stages, respectively) higher levels of saliva- and FSG-specific IgG1 antibodies than resistant hosts (Fig. 2a, b). In all, these findings indicate that, in susceptible animals, levels of tick-specific antibodies begin to correlate with tick loads only after successive infestations. They also indicate that upon primary and subsequent exposures to ticks, resistant animals maintain consistent levels of antibodies specific for saliva and FSG.

**Fig. 3:**
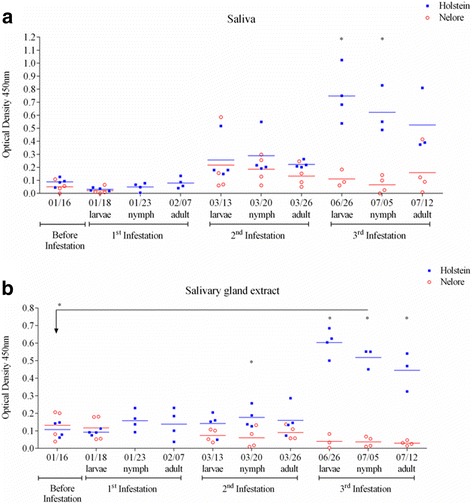
Levels of IgG2 antibodies specific for saliva and extracts of salivary glands from female ticks differ between tick-resistant and tick-susceptible breeds of cattle. Levels of IgG2 antibodies specific for saliva (**a**) and for salivary gland extracts from female ticks (**b**) were measured in sera from animals of a tick-susceptible (Holstein, *blue squares*) or tick-resistant (Nelore, *red open circles*) breed of cattle. Antibodies were measured before (baseline levels) and during three successive artificial infestations with ticks, when the parasites were at the larval, nymphal and adult stages of their life-cycle. A dilution of 1:100 was used for each serum. Asterisks indicate the levels of significance between amounts of specific IgG2 antibodies in Holstein and Nelore hosts and the specific statistical results are described in the text

Resistant and susceptible hosts presented with similar levels of saliva- and FSG-specific IgG2 antibodies before and during the first two artificial infestations (Fig. 3a, b). Relative to baseline levels, during the third infestation FSG-specific IgG2 antibodies increased significantly (ANOVA, *F*_(1.828, 3.656)_ = 22.30, *P* = 0.0092) in susceptible hosts undergoing infestations with nymphs (Fig. 3b). In addition, by the third infestation and during all developmental stages of ticks, susceptible hosts presented with significantly (*t*-test, *t*_(11.43)_ = 5, *P <* 0.0001, *t*_(14.67)_ = 5, *P <* 0.0001 and *t*_(7.723)_ = 5, *P* = 0.0006, respectively for the larval, nymphal and adult stages) higher levels of FSG-specific IgG2 antibodies when compared with resistant hosts (Fig. 3b). Similar differences were seen between levels of saliva-specific IgG2 antibodies in resistant and susceptible bovines (Fig. 3a).

In summary, upon the first exposure to ticks, resistant bovines present significantly higher levels of female salivary protein-specific IgG1 antibodies, but not of IgG2 antibodies when compared with susceptible bovines undergoing the same level of exposure. Conversely, successive infestations in susceptible bovines are accompanied by a continual increase in the levels of female salivary protein-specific IgG1 and IgG2 antibodies.

### Antibodies from tick-resistant hosts recognize a larger repertoire of tick salivary proteins than antibodies from susceptible hosts

**Fig. 4:**
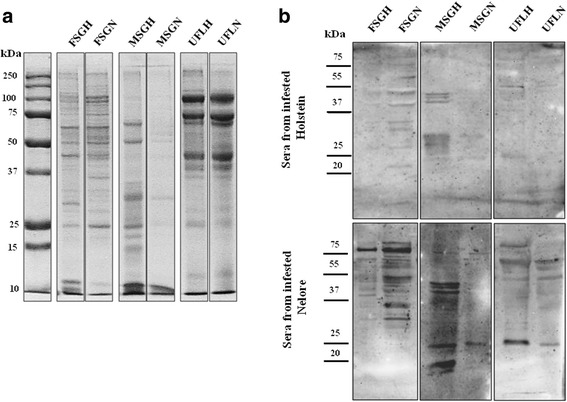
Sera from twice-infested, genetically tick-resistant breeds of bovines react with more tick salivary proteins than sera from genetically tick-susceptible bovines. **a** Protein extracts of FSG, MSG and UFL (7 μg of each) from ticks fed on resistant (R) and susceptible (H) hosts were separated in 12% SDS-PAGE gels and then stained with Coomassie blue. Molecular weight markers (kDa) are indicated on the left of the gel. **b** Sera were reacted in protein blots of extracts of FSG, MSG and UFL of *R. microplus* separated by electrophoresis in one dimension. Sera were pooled from twice-infested, tick-susceptible (Holstein breed) or tick-resistant (Nelore breed) bovines (*N* = 4 of each) when infested with larvae, nymphs and adults, totaling 12 sera in each pool) and reacted with proteins from the indicated extracts. The end dilution used was 1:75. *Abbreviations*: FSGH, extract of salivary glands from female ticks fed on Holsteins; FSGN, extract of salivary glands from female ticks fed on Nelores; MSGH, extract of salivary glands from male ticks fed on Holsteins; MSGN, extract of salivary glands from male ticks fed on Nelores; UFLH, extract of larvae hatched from egg masses laid by female ticks fed on Holsteins; UFLN, extract of larvae hatched from egg masses laid by female ticks fed on Nelores

**Fig. 5:**
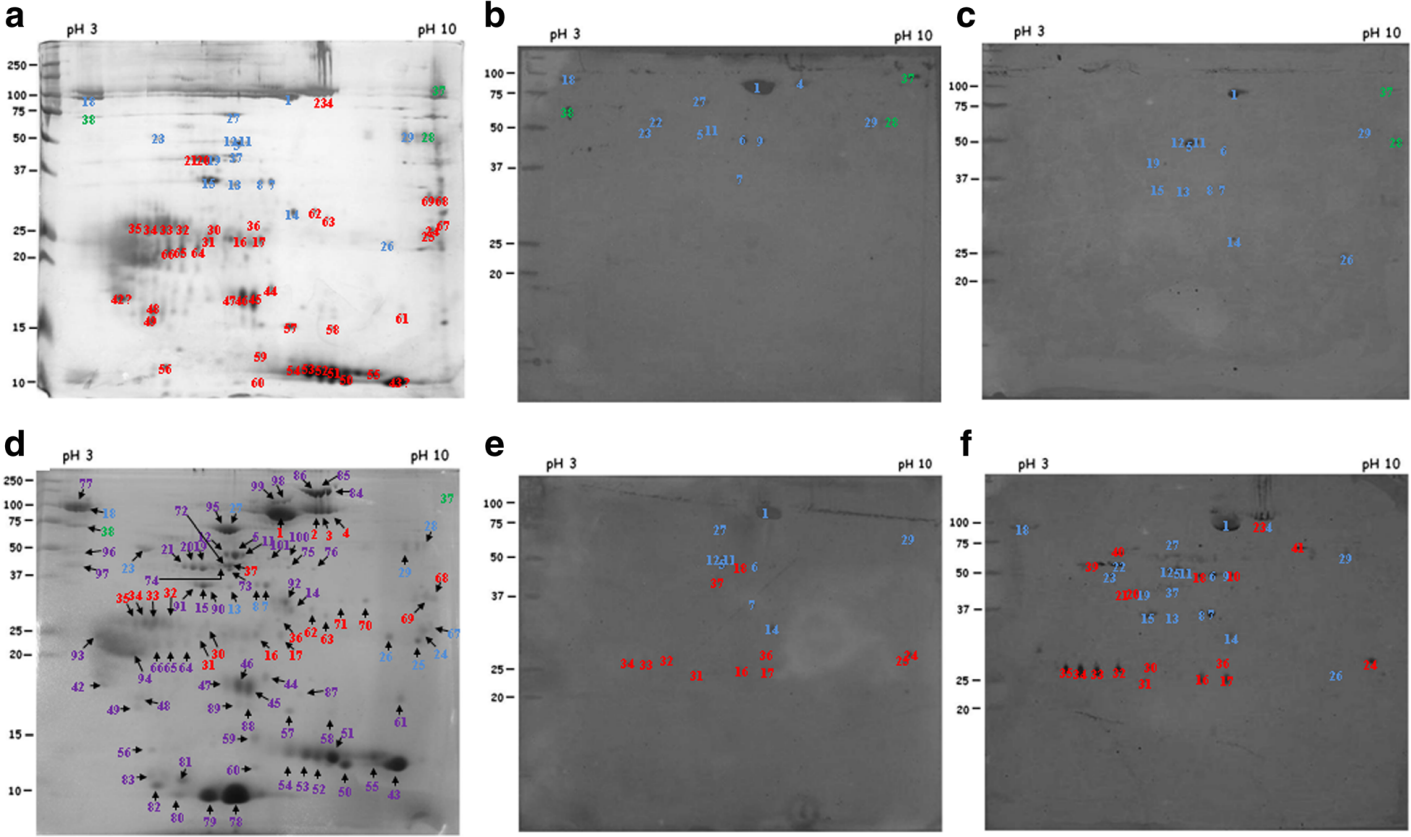
Identification of salivary proteins from *R. microplus* that react with sera from tick-susceptible and tick-resistant bovines. A pool of saliva collected from female ticks feeding on genetically susceptible hosts was focalized on 13 cm pH3-10 L (left to right) strips in the first dimension and 12% SDS-PAGE gels in the second dimension. Molecular weight markers are indicated on the left (kDa). **a** Gel stained with silver. **b**-**f** Gels run in parallel with the gel shown in (**a**) were transferred to nitrocellulose membranes, incubated with pooled sera diluted 1:100 from susceptible (Holstein breed: **b** and **c**) or resistant (Nelore breed: **e** and **f**) hosts, before (non-infested) and after infestation (larva, nymph and adult stages), respectively, and developed with protein-G conjugated with peroxidase (diluted 1:2000). Reactive spots are highlighted (**d**) and were excised separately and analyzed by MS. Results are shown in Table 1

While levels of immunoglobulins and of saliva-specific antibodies differ between the breeds of bovine hosts and are associated with the size of the tick loads, the specificities of the antibodies for individual proteins are not known. We thus examined the specificities of antibodies produced by the two types of bovine hosts to tick salivary proteins resolved in one (Fig. 4) and two dimensions (Fig. 5). Figure 4a presents the complete profiles of proteins obtained with extracts of female and male salivary glands and of unfed larvae originating from ticks fed on susceptible or resistant hosts resolved in one dimension; Fig. 4b presents the profiles of reactivity against these tick proteins with antibodies from resistant and susceptible bovines undergoing all developmental stages of the second successive infestation with *R. microplus*. The profiles show that, in spite of presenting lower tick loads and lower levels of IgG1 and IgG2 antibodies specific for extracts of salivary proteins, sera from animals of the tick-resistant breed (Nelores) recognize a much larger range of tick proteins, some of which with greater intensity than sera from susceptible hosts (Fig. 4b). Interestingly, we also observed that reactivity profiles of sera with extracts of salivary glands from ticks fed on either resistant or susceptible hosts differed, indicating that the composition of these extracts also varies and, therefore, that ticks modify their salivary protein repertoire according to the host that they feed on (Fig. 4a).

### Immunoblot analyses of *R. microplus* salivary antigens separated in two dimensions: sera from resistant bovine hosts react with a broader range of proteins related to putative functions of parasitism

We identified tick salivary proteins that are differentially recognized by the two types of host by resolving in two dimensional electrophoresis saliva from female ticks feeding on tick-susceptible bovine hosts, followed by spot picking and sequencing by LC-MS and running in parallel gels for immunoblots with informative the sera. We succeeded in identifying proteins from a total of 101 spots in 2-D gels (Additional file 2: Table S2). Of these spots, 64 were reactive with at least one group of pooled sera and 55 did not react with sera from any group. The results presented in Fig. 5 and Table 1 show that 8 spots were recognized by all groups of sera; putative functions of proteins identified were those of a Kunitz inhibitor, a serpin, a tropomyosin and cathepsin D2, among others.

**Table 1:** Salivary proteins of female *R. microplus* ticks recognized by sera from naïve and/or two-times infested genetically tick-susceptible and/or tick-resistant bovines

Spot No. in figure	CDS in sialotranscriptome^a^	Annotation and putative function of protein	MW Kda^b^	Source of pooled reactive sera^c^
34	20757	14-3-3 CG17870-PA, isoform A isoform 2	27	NR IR
33	20757	14-3-3 CG17870-PA, isoform A isoform 2	27	NR IR
20	18420	actin	42	IR
21	18420	actin	42	IR
19	18420	actin	42	IR IS
22	18420	actin	42	IR NS
1	109972	anticoagulant protein rhipilin-1	17	NR IR NS IS
37	31546	apolipophorin	89	NR IR
18	31546	apolipophorin	89	NR IR
22	7197	beta tubulin	53	IR NS
22	22306	beta tubulin	50	IR NS
22	22310	beta tubulin	35	IR NS
29	14491	cathepsin D2	42	NS IS NR IR
20	39114	cathepsin L-like cysteine proteinase B	38	IR
21	39114	cathepsin L-like cysteine proteinase B	38	IR
19	39114	cathepsin L-like cysteine proteinase B	38	IR IS
1	50205	CBF1-interacting corepressor	30	NR IR NS IS
40	30215	chaperonin subunit	65	IR
9	111414	CRISP3-cysteine-rich secretory protein	46	IR NS
30	25106	enolase	43	IR
24	67998	ENSANGP00000022132	25	NR IR
22	4388	F0F1-type ATP synthase, beta subunit	59	IR NS
25	111630	glutathione S-transferase	31	NR
24	111630	glutathione S-transferase	31	NR IR
38	21956	heat shock protein	71	NS
37	4142	heavy-chain filboin	44	NR IR
14	71787	hypothetical protein	22	NR IR IS
1	110513	hypothetical protein	3	NR IR NS IS
38	121267	hypothetical protein BRAFLDRAFT_287019	45	NS
20	122308	longipain cystein protease	33	IR
21	122308	longipain cystein protease	33	IR
19	122308	longipain cystein protease	33	IR IS
13	113489	lospin 8 type serpin	43	IR IS
12	113489	lospin 8 type serpin	43	NR IR IS
11	113489	lospin 8 type serpin	43	NR IR NS IS
6	113489	lospin 8 type serpin	43	NR IR NS IS
7	113489	lospin 8 type serpin	43	NR IR NS IS
10	113489	lospin 8 type serpin	43	IR
8	113489	lospin 8 type serpin	43	IR IS
5	113489	lospin 8 type serpin	43	NR IR IS
11	171727	lysosomal acid phosphatase	26	NR IR NS IS
40	9166	protein disulfite isomerase-2	39	IR
40	10534	protein disulfite isomerase-2	39	IR
12	39744	putative chitinase	45	NR IR IS
15	128399	putative salivary secreted protein	36	IR IS
24	83247	putative salivary secreted protein	25	NR IR
14	77570	putative salivary secreted protein	25	NR IR IS
14	85307	putative salivary secreted protein	27	NR IR IS
35	108605	putative secreted protein	24	IR
35	171393	putative secreted protein	18	IR
32	171393	putative secreted protein	18	NR IR
33	171393	putative secreted protein	18	NR IR
34	171393	putative secreted protein	18	NR IR
30	174664	putative secreted protein (histamine-binding?)	21	IR
31	174664	putative secreted protein (histamine-binding?)	21	NR IR
32	174664	putative secreted protein (histamine-binding?)	21	NR IR
33	174664	putative secreted protein (histamine-binding?)	21	NR IR
34	174664	putative secreted protein (histamine-binding?)	21	NR IR
31	178127	putative secreted protein (histamine-binding?)	21	NR IR
33	178127	putative secreted protein (histamine-binding?)	21	NR IR
34	178127	putative secreted protein (histamine-binding?)	21	NR IR
35	178127	putative secreted protein (histamine-binding?)	21	IR
14	54776	putative thyropin precursor	23	NR IR IS
17	113785	salivary lipid interacting protein	20	NR IR
36	113785	salivary lipid interacting protein	20	NR IR
16	113785	salivary lipid interacting protein	20	NR IR
8	38904	secreted protein	38	IR IS
31	164102	secreted protein	23	NR IR
31	164103	secreted protein	23	NR IR
33	164103	secreted protein	23	NR IR
7	38904	secreted protein	38	NR IR NS IS
16	128399	secreted protein	36	NR IR
17	128399	secreted protein	36	NR IR
26	8103	secreted salivary gland peptide	25	IS IR
25	8103	secreted salivary gland peptide	25	NR
24	8103	secreted salivary gland peptide	25	NR IR
27	8103	secreted salivary gland peptide	25	NR IR NS
24	26121	selenium-dependent salivary glutathione peroxidase	18	NR IR
20	106322	serine proteinase inhibitor serpin-3	43	IR
21	106322	serine proteinase inhibitor serpin-3	43	IR
19	106322	serine proteinase inhibitor serpin-3	43	IR IS
30	106322	serine proteinase inhibitor serpin-3	43	IR
20	106321	serine proteinase inhibitor serpin-3	43	IR
19	106321	serine proteinase inhibitor serpin-3	43	IR IS
12	6955	serpin-2 precursor	36	NR IR IS
28	13343	translation elongation factor EF-1 alpha-Tu	51	NS IS
29	13343	translation elongation factor EF-1 alpha-Tu	51	NS IS NR IR
31	32190	uncharacterized protein	17	NR IR
37	43507	vitellogenin-1	158	NR IR
18	43508	vitellogenin-1	97	NR IR
18	43509	vitellogenin-1	182	NR IR

*Note*: The table is formatted according to annotation of putative function of protein, but not by spot number recovered from gel. In some instances there are more than one protein identified in the spot collected

^a^CDS: coding sequences generated by the sialotranscriptome of *R. microplus* and deposited at GenBank - NCBI (BioProject ID PRJNA329522)

^b^Molecular weight furnished by Scaffold software

^c^ *Abbreviations* for sources of sera and samples: NR, pooled sera collected from four tick-naïve, genetically resistant Nelore; IR, pooled sera from four genetically resistant, twice-infested Nelore bovines at the end of the infestation; NS, pooled sera collected from four tick-naïve, genetically susceptible Holstein bovines; IS, pooled sera from four genetically susceptible, twice-infested Holstein bovines at the end of the infestation

Salivary proteins were obtained from saliva of female *R. microplus* ticks feeding on genetically susceptible hosts. Sera employed were obtained from naïve and/or two-times infested genetically tick-susceptible and/or tick -resistant bovines. Criteria employed to identify each sequence: minimum of one peptide presenting with 90% probability of being the protein and 90% probability of being the peptide

Pooled sera from tick-resistant Nelores reacted with twice the number of spots than pooled sera from tick-susceptible Holsteins, 39 *vs* 21 spots, respectively (Fig. 5 and Table 1). Among putative functions of proteins in spots reacting exclusively with sera from tick-resistant Nelores were an apolipophorin, a salivary lipid interacting protein, histamine-binding proteins, protein disulfide isomerases, serpin-3, and vitellogenins.

Many proteins encoded by the same CDS (BioProject ID PRJNA329522) were present in more than one spot, but were recognized by sera from either resistant or susceptible bovines. For example, a cathepsin L-like cysteine proteinase B and a longipain, encoded by CDS39114 and CDS122308, respectively, were both found in three distinct spots, two of which reacted exclusively with pooled sera from infested Nelores and one with sera from infested Holsteins and Nelores.

Among the spots that reacted exclusively with sera from susceptible, infested bovines was one that contained a glutathione S-transferase. Another glutathione S-transferase encoded by a distinct CDS reacted exclusively with sera from both naïve and infested Nelores, being that the enzyme recognized by Nelore sera presented a pI and a MW that differed from that of the functionally similar enzyme recognized by sera from susceptible bovines.

## Discussion

Cattle ticks are able to remain on their bovine hosts for approximately three weeks and during this period they ingest relatively large amounts of blood containing antibodies. Many of these antibodies are potentially specific for tick saliva, especially in repeatedly infested hosts. The present study examined antibody responses to *R. microplus* ticks in a model of bovines presenting contrasting phenotypes of infestations and undergoing artificial infestations: it examines levels of total IgG1, IgG2 and IgE as well as the specific antibody responses and profiles of reactivities to different sets of tick salivary antigens.

We first showed that levels of total IgG1 and IgG2 immunoglobulins are higher in the susceptible breed before and during infestations. This finding concurs with the observations made by Rechav [37], who followed immunoglobulin levels for thirty-six months and showed that concentrations of gamma globulins were consistently higher in bovines of the tick-susceptible, Hereford taurine breed than in the tick-resistant, Brahman indicine breed. Rechav [37] also found a positive correlation between the number of adult ticks removed from hosts and levels of immunoglobulins. In the present study, during the first infestation, levels of total IgG1 and IgG2 decreased in tick-susceptible Holsteins, but did not change significantly in tick-resistant Nelore hosts. The decrease seen in Holsteins cannot be attributed to the action of tick salivary IgG-binding proteins since it was patent two days after larvae were released and this mediator of parasitism is produced solely by male ticks [40], including in *R. microplus* [32]. On the other hand, tick infestations cause stress to their hosts [41, 42] and levels of cortisol are known to be inversely related to levels of serum immunoglobulins in bovines [43]. Levels of serum immunoglobulins are also inversely correlated with their half-life due to the phenomenon of concentration-catabolism, in which the availability of the IgG salvage receptor (or neonatal Fc receptor, FcRn) determines the serum concentration of IgG [44, 45]. Of relevance to this study, functional FcRn is produced by keratinocytes in skin [46, 47] and is detected in epithelial cells of the hair follicles and sebaceous glands and in melanocytes [47]; in phagocytic cells it can be upregulated by TNF-alpha [48] and downregulated by IFN-gamma [49]. Furthermore, tick infestations induce acute phase responses [41], in which the levels of a large set of proteins increase or decrease. Some of these proteins have been shown to bind to immunoglobulin Fc receptors of phagocytic leukocytes [50] and we speculate that they may affect levels of IgGs by altering the availability of salvage FcRn or catabolic FcR.

Regarding levels of total IgE, these did not differ between bovine breeds examined in this study. However, they were affected by tick infestations in both breeds: they increased during successive infestations, but by the third infestation had returned to basal levels. Once explanation for this finding is the fact that IgE has a shorter half-life than IgG and the observed decline thus results from the rapid catabolism of this class of antibody. It is also possible that this decrease was caused by recruitment of homocytotropic IgE antibodies to the site of tick attachment and/or recruitment of IgE secreting-cells in this site. Indeed, IgG is found deposited near tick attachment sites in the skin of tick-resistant guinea-pigs [51].

Beekeepers develop immune tolerance by after repeated exposure to bee venom, which is also delivered through the skin [52]: in spite of detectable levels of venom-specific IgE, titers of venom-specific IgG4 antibodies reach extremely high levels at the end of the beekeeping season and are positively correlated with levels of venom antigen-specific T regulatory cells, which increase upon persistence of the antigen and return to initial levels within a few months after individuals are no longer exposed to stings. The equivalent of allergen-blocking IgG4 antibodies has not been described in cattle, but it is intriguing that in spite of high levels of challenge with tick saliva, levels of IgE decreased during the third round of infestation.

Regarding levels of saliva and FSG extract-specific IgG1 and IgG2 antibodies, upon the first exposure to ticks, resistant bovines present significantly higher levels of IgG1 antibodies, but not of IgG2 antibodies, compared with susceptible bovines undergoing the same level of exposure and, after subsequent exposure, resistant animals maintain the same levels of antibodies. Successive infestations in susceptible bovines were accompanied by a continual increase in the levels of IgG1 and IgG2 antibodies specific for saliva or for extracts of salivary glands from female ticks.

Successive infestations in C3H/HeJ mice polarize the T helper cellular response towards a TH2 phenotype, but the profile of corresponding antibody responses in that situation was not examined [53]. Nevertheless, in the present work the antibody profiles in both breeds of bovines do not point towards a polarized helper T lymphocyte response because levels of saliva and SGE-specific IgG1 and IgG2 antibodies were similar. Menten-Dedoyart and colleagues [54] examined antibody responses specific for bovine serum albumin (BSA) made by BALB/c mice artificially infested with a single couple of adult *Ixodes ricinus* before or after immunization with this antigen. They observed that infestations did not affect the ratio of IgG1 to IgG2 anti-BSA antibodies. These authors also demonstrated that infestations inhibited production of antibodies by plasma cells, but not the development of B memory cells since recall antibody responses were similar to those seen in non-infested, control mice [55].

A study by Piper and colleagues [27] examined differences in antibody responses mounted by tick-resistant Brahmans and susceptible Holsteins that were infested artificially and had been exposed to ticks since birth. In contrast to the results obtained in the present study, Piper et al. [27] found that IgG1, but not IgG2 tick-specific antibodies were significantly higher in susceptible hosts than in resistant hosts. This discrepancy may be caused by differences in the experimental designs of the two studies: our study employed whole saliva from female ticks as the antigen for measuring antibodies and not two types of fractions from extracts of salivary glands (membrane-bound and soluble); in addition Piper et al. [27] did not describe the origin of the anti-bovine IgG1 and IgG2 antibodies employed in their assays and differences in IgG allotypes recognized by the reagents from different manufacturers may affect results from each laboratory. Previous work by some of us [25] showed that the IgG1 and IgG2 antibody responses to tick saliva in naturally infested bovines were significantly decreased in susceptible hosts when pastures were highly infested compared to these same hosts when pastures were lightly infested. Levels of these antibodies remained stable in resistant hosts regardless of the intensity of infestation of the pasture. In that study cattle were exposed to thousands of larvae produced by hundreds of females dropped from the hosts; in the case of the present study hosts were infested with 10,000 larvae produced by a few females.

We also sought to identify the proteins recognized differentially by the two types of host. We succeeded in identifying proteins from a total of 101 spots from saliva in 2-D gels followed by LC-MS. Many of the proteins identified in our immunoproteome have already been identified in a salivary proteome of *R. microplus* [56], as well as in those of other species of ticks [57–60]. Tirlone et al. [56] compared the profile of saliva of partially and fully engorged females of *R. microplus* fed on Hereford calves; the proteomic analysis employed 1D gel electrophoresis and LC-MS/MS. They identified 187 different tick salivary proteins, as well as 68 proteins from the bovine host [56]. The authors also showed that protein profile of saliva changes according the stage of feeding and identified proteins that were found exclusively in partially or fully engorged females and others that were shared between these stages. Similar findings were obtained with *Haemaphysalis longicornis* ticks fed on rabbits, where 135 proteins were present in the saliva of both nymph and female ticks, of which 30 proteins were identified exclusively in fully saliva of engorged nymphs, 74 in saliva of fully engorged females, and 31 were detected in both instars [57]. Oliveira et al. [58] compared the protein profile of saliva from *Rhipicephalus sanguineus* female ticks fed on rabbits which had been collected with two salivation stimulants (dopamine and pilocarpine) and proteomic analysis employed 1D gel electrophoresis followed by reversed-phase HPLC and MS/MS analysis. Their study showed that saliva obtained with pilocarpine presented with more proteins than when obtained with dopamine; however, few proteins associated with parasitism and blood-feeding were identified. A similar study using salivary glands from female *Amblyomma variegatum* ticks fed on *B. indicus* was performed using 1D gel electrophoresis followed by nanoflow reverse-phase liquid chromatography tandem mass spectrometry [60]; this study also identified only a few tick proteins that could be associated with parasitism. Díaz-Martín et al. [59] performed a proteomic study comparing saliva from females and males of *Ornithodoros moubata* ticks with LC-MS/MS. They identified 193 proteins and there was a notable difference in the proteomes of females and males, with few proteins shared by both sexes. On the other hand, our proteomic approach employing 2D gel followed by mass sequencing was able to identify a larger number of proteins associated with parasitism and hematophagy when compared with 1D gel approaches followed by mass sequencing. Moreover, the saliva used in these other proteomic studies were obtained from different species of ticks and developmental stages of ticks as well as ticks fed on different hosts, resulting in different protein profiles. Nevertheless, we observed that these different saliva presented numerous proteins in common including several conserved and structural proteins (actin, tubulin and others), besides salivary proteins that potentially participate in parasitism, such as those containing Kunitz or ML domains, antimicrobial peptides, serpins, lipocalins, cystatins, tryropins, glycine-rich proteins, mucins, chitinases, disulfide isomerase cathepsin, glutathione peroxidases, vitellogenin and others [56–60]. However, none of these previous proteomic studies identified neutrophil elastase inhibitors as we have found in the present study.

In the present report, among the 101 proteins identified, 8 spots were recognized by all groups of sera; putative functions of proteins identified were those of a Kunitz inhibitor, a serpin, a tropomyosin and cathepsin D2, among others. Noteworthy was the finding that tick-resistant Nelores reacted with twice the number of spots than pooled sera from tick-susceptible Holsteins, 39 *versus* 21 spots, respectively. Among putative functions of proteins in spots reacting exclusively with sera from tick-resistant Nelores were an apolipophorin, a salivary lipid interacting protein, histamine-binding proteins (i.e. lipocalins), protein disulfide isomerases, serpin-3, and vitellogenins. Interestingly, as pointed out above, these proteins are always present in salivary proteome studies of different species of ticks [56–60], so they can be considered good targets to compose a vaccine against multiple species of ticks.

Insect apolipoproteins are homologous to mammalian apolipoprotein E, which is involved in LPS detoxification, phagocytosis and pattern recognition. Indeed, an insect apolipophorin has been shown to participate in recognition of beta-1,3-glucan molecular patterns and cellular encapsulation reactions [61]. The lipid interacting protein possesses an MD-2-related lipid-recognition (ML) domain that plays a role in the recognition of pathogens. This domain is also present in proteins from other arthropod pests, such as in the house-dust mite allergen proteins such as Der f 2 from *Dermatophagoides farinae* and Der p 2 from *D. pteronyssinus* [62]. The lipocalins as histamine-binding proteins have been found extensively in the transcriptome (BioProject ID PRJNA329522) and proteomes [56] from *R. microplus* ticks and other ticks [57–60, 63–65]. These proteins may remove histamine generated in local itching reactions to tick bites and this function may neutralized by antibodies and, thus, compromised in resistant bovine hosts. Indeed, Willadsen et al. [66] have proposed that the amount of histamine available locally in the skin may have a role in the resistance to ticks. Protein disulfide isomerases play a role in guiding correct protein folding through formation and breakage of disulfide bonds and chaperone activity. Silencing of these enzymes in ticks affects blood-feeding and oviposition and overall tick viability [67]. Thus, neutralization of these salivary proteins by antibodies may explain the poor outcome of the life-cycle of *R. microplus* in Nelore hosts. In accordance with results presented herein, Rodriguez-Valle and colleagues [68] showed that RMS-3, a serpin from *R. microplus*, reacts more intensely with sera from genetically resistant bovine hosts [68]. The vitellogenins recognized exclusively by sera of tick-resistant hosts are also representatives of apoliproteins and contain lipid-binding domains, furthermore they participate in fertility of female ticks [69].

Many proteins encoded by the same CDS (BioProject ID PRJNA329522) were present in more than one spot and were individually recognized by different groups of sera. These patterns of reactivity may reflect that bovine hosts of different genetic compositions react with different post-translational modifications. Conversely, they may reflect different levels of antibodies between these groups of hosts, consequently affecting the sensibility of the immunoblotting assay. Among the spots that reacted exclusively with sera from susceptible, infested Holsteins was one that contained glutathione S-transferase, an enzyme that controls detoxification processes. Apparently, neutralization of these and other proteins by antibodies induced in genetically susceptible Holsteins does not confer significant advantages towards diminishing tick loads in these hosts. Another glutathione S-transferase encoded by a distinct CDS reacted exclusively with sera from Nelores, from both naïve and twice-infested animals. The significance of this finding in terms of resistance to ticks is not clear, but the enzymes recognized by Nelore sera presented a pI and a MW that differed from that of the enzyme recognized by sera from Holsteins.

In all, antibodies generated by Nelores before and after they were infested tended to react with proteins directly involved in mechanisms of parasitism and parasite escape mechanisms. In addition, antibodies generated by Nelores reacted with a greater number of tick salivary proteins. Indicine cattle may thus bear significantly lower tick loads because they neutralize more efficiently functions of tick saliva. Regarding the non-reactive components present in tick saliva and salivary gland extracts, Kotsyfakis et al. [70] have shown that these proteins can be rendered immunogenic with an adjuvant and that the elicited immune response subsequently affects tick biology. This indicates that these immunologically silent antigens are important in parasitism. The fact that some salivary proteins are immunogenic without exogenous intervention in some genetic backgrounds raises questions about the mechanisms behind this phenomenon. As mentioned previously herein, soluble proteins are immunogenic when they are aggregated or delivered with adjuvants [23, 24]. Genetically tick-resistant hosts are either able to aggregate more tick salivary proteins and/or are more responsive to some form of signaling *via* components of innate immunity. Th2 responses to an antigen administered epicutaneously, the situation that occurs during tick infestations, are downregulated by the cleavage product of C3 convertase, C3a, produced at the site of injury to the skin [71]. Saliva from *R. microplus* ticks [72] can inhibit activation of complement, however to date a specific inhibitor of C3 convertase has been described in prostriate ticks [73], but not in *R. microplus*. In addition, the data described herein (see Figs. 2 and 3) do not indicate that antibody responses are polarized towards a specific phenotype of T lymphocytes. However, assays that measure levels of IgG1 and IgG2 antibodies specific for individual tick salivary antigens must be performed to confirm this finding.

Besides these possibilities, one must also consider that the balance of tick salivary components differs in ticks feeding on resistant and susceptible hosts (Fig. 4a) [58, 61]. In both cases, several of these components have the potential to affect outcome of antigen recognition, processing and presentation, as well as assembly of effective immunity. For example, serpins affect assembly and outcome of immune responses mediated by T lymphocytes, including memory responses, by inhibiting enzymes of antigen processing and of cellular death programs. Importantly, pathogen-derived serpins also can inhibit the proteases involved in these processes (reviewed by Ashton-Rickardt [74]). As shown by us (BioProject ID PRJNA329522) and others [75–77], salivary glands of *R microplus* contain a large repertoire of serpins and their role in modulating adaptive immune responses warrants further investigation. In addition, some tick salivary proteins tend to aggregate other proteins and to bind salivary proteins to host skin [78]. This has been shown to depend on an esterase and on a protease inhibitor. We have shown in this report that many protease inhibitors are well represented in tick saliva and in another report (BioProject ID PRJNA329522) that their rate of transcription differs according to the genetic background of the host that ticks feed on.

## Conclusions

William Trager [79] was the first to show that sera passively transferred from tick-infested to non-infested guinea pigs subsequently challenged with ticks affected the infestation by decreasing engorgement of the ticks feeding on those hosts. Passive transfer of plasma from infested to non-infested hosts will only confer resistance to challenge infestations if the plasma is derived from genetically resistant animals [80]. These results show that there are qualitative and quantitative differences in humoral immune responses between susceptible and resistant hosts of ticks. In the present work, we measured the levels of total IgG1, IgG2 and IgE immunoglobulins and of saliva- and FSG-specific IgG1 and IgG2 antibodies in sera from tick-resistant and tick-susceptible host that were successively infested three times, followed by identification of tick antigens recognized differentially by sera from twice-infested hosts. The results show that susceptible animals have significantly higher levels of total IgG1 and IgG2 immunoglobulins than resistant hosts after successive infestations. On the other hand, susceptible hosts modulated levels of total IgG immunoglobulins and saliva- and FSG-specific IgG1 antibodies when infested for the first time with ticks. The innate humoral immune response that resistant hosts present upon a primary exposure to larvae may contain the infestations and increase the immunogenicity of salivary components. In humans, natural antibodies appear to be important in the immune responses against pathogens, since they have a high affinity for carbohydrate groups present in the membrane of many pathogens [81] and tick salivary proteins are glycosylated [82]. Another finding in this study is that, at the second successive infestation, the levels of total IgE increased in sera from both susceptible and resistant hosts and then decreased significantly at the third infestation. It is possible that this decrease was caused by recruitment of homocytotropic IgE antibodies to the site of tick attachment and/or recruitment of IgE secreting-cells in this site. A study that strengthens this hypothesis showed that IgG is found deposited near tick attachment sites in the skin of tick-resistant guinea-pigs [51]. Tolerance to tick salivary proteins, such as occurs against bee venom [52], is another possibility. Finally, our findings indicate that antibodies are related with resistance to ticks provided they react with a large repertoire of tick salivary proteins. The reasons for the greater immunogenicity of tick salivary proteins in genetically resistant hosts and/or the greater immunosuppressive capacity of tick saliva for susceptible hosts must now be investigated.

## Additional files

Additional file 1: Table S1. Sera used in ELISA and proteome analyses. (DOCX 39 kb)
Additional file 2: Table S2. Identification of all spots recovered and sequenced from immunoblotting analyses using two-dimensional gel electrophoresis. (DOCX 57 kb)

## Abbreviations

DTT: Dithiotreitol
EDTA: Ethylenediaminetetraacetic acid
ELISA: Enzyme-linked immunosorbent assay
FBS: Fetal bovine serum
FSG: Female salivary gland extract
FSGR: Female salivary gland from ticks fed on resistant Nelores
FSGS: Female salivary gland from ticks fed on susceptible Holsteins
IgG: Immunoglobulin G
IgGBPs: Immunoglobulin G-binding proteins
LC-MS: Liquid chromatography - mass spectrometry
MSG: Male salivary gland extract
MSGR: Male salivary gland from tick fed on resistant Nelores
MSGS: Male salivary gland from tick fed on susceptible Holsteins
PBS: Phosphate buffered saline
PVDF: Polyvinylidene fluoride
TBS-T: TRIS buffered saline-tween 20
TMB: 3,3′,5,5′-tetramethylbenzidine
UFL: Extract of unfed larvae
UFLR: Extract of unfed larvae originated from female ticks fed on resistant Nelores
UFLS: Extract of unfed larvae originates from female ticks fed on susceptible Holsteins
