# Supplementary material for: Immune recognition of salivary proteins from the cattle tick Rhipicephalus microplus differs according to the genotype of the bovine host
Source: Parasit Vectors. 2017 Mar 14;10:144. doi: 10.1186/s13071-017-2077-9 (PMC5348738; doi:10.1186/s13071-017-2077-9)
Supplement: Additional file 1: Table S1. — Sera used in ELISA and proteome analyses. (DOCX 39 kb) [file 13071_2017_2077_MOESM1_ESM.docx]

**Additional file 1.** Sera used in ELISA and proteome analyses.

| **Breed of bovine host/**  **Number of unfed larvae infesting host** | **Number of**  **infestations** | **Developmental stage of tick when sera were sampled/**  **(n = number of bovines sampled)** | | | |
| --- | --- | --- | --- | --- | --- |
|  |  | **Before infestation** | **Larva** | **Nymph** | **Adult** |
| Holstein/  10,000 | 1 | 4 | 4 | 4 | 4 |
|  | 2 |  | 4 | 4 | 4 |
|  | 3 |  | 4 | 3^†^ | 3^†^ |
| Nelore/  20,000 | 1 | 4 | 4 | ND* | ND* |
|  | 2 |  | 4 | 4 | 4 |
|  | 3 |  | 3# | 4 | 4 |

Description of sera samples from bovines artificially infested with unfed larvae of *R. microplus*, in which amounts of immunoglobulins and levels of tick salivary protein-specific antibodies were evaluated with immunoenzymatic assays (assayed individually) and in which reactivities were assessed with immunoblots of tick salivary proteins separated by electrophoreses in one and in two dimensions (assayed as pooled sera from hosts before infestations and from twice-infested hosts). **^*^** The sera of first infestation from Nelore were not collected in stages of the nymphs and adults due to technical problems. ^#^ The serum from an animal (Nelore) was not collected due to technical problems. ^†^ The third infestation an animal (Holstein) died during the experiment.
