# Supplementary material for: Immune recognition of salivary proteins from the cattle tick Rhipicephalus microplus differs according to the genotype of the bovine host
Source: Parasit Vectors. 2017 Mar 14;10:144. doi: 10.1186/s13071-017-2077-9 (PMC5348738; doi:10.1186/s13071-017-2077-9)
Supplement: Additional file 2: Table S2. — Identification of all spots recovered and sequenced from immunoblotting analyses using two-dimensional gel electrophoresis. (DOCX 57 kb) [file 13071_2017_2077_MOESM2_ESM.docx]

**Additional file 2.** Identification of all spots recovered and sequenced from Immunoblotting analyses using two-dimensional gel electrophoresis.

| **Spot N^o^ in Figure** | **CDS in Sialotranscriptome^1^** | **Annotation and Putative Function of Protein** | **MW kDa^2^** | **Source of Pooled Reactive Sera^3^** |
| --- | --- | --- | --- | --- |
| 1 | 109972 | anticoagulant protein rhipilin-1 | 17 | NS IS NR IR |
|  | 50205 | CBF1-interacting corepressor | 30 |  |
|  | 110513 | hypothetical protein | 3 |  |
| 5 | 113489 | lospin 8 | 43 | IS NR IR |
| 6 | 113489 | lospin 8 | 43 | NS IS NR IR |
| 7 | 113489 | lospin 8 | 43 | NS IS NR IR |
|  | 38904 | secreted protein | 38 |  |
| 8 | 113489 | lospin 8 | 43 | IR IS |
|  | 38904 | secreted protein | 38 |  |
| 9 | 111414 | CRISP3- cysteine-rich secretory protein | 46 | NS IR |
| 10 | 113489 | lospin 8 | 43 | IR |
| 11 | 113489 | lospin 8 | 43 | NR IR NS IS |
|  | 171727 | lysosomal acid phosphatase | 26 |  |
| 12 | 39744 | putative chitinase | 45 | NR IR IS |
|  | 6955 | serpin-2 precursor | 36 |  |
|  | 113489 | lospin 8 | 43 |  |
| 13 | 113489 | lospin 8 | 43 | IR IS |
| 14 | 77570 | putative salivary secreted protein | 25 | NR IR IS |
|  | 71787 | hypothetical protein | 22 |  |
|  | 54776 | putative thyropin precursor | 23 |  |
|  | 85307 | putative salivary secreted protein | 27 |  |
| 15 | 128399 | putative salivary secreted protein | 36 | IR IS |
| 16 | 128399 | secreted protein, putative | 36 | NR IR |
|  | 113785 | salivary lipid interacting protein, putative | 20 |  |
| 17 | 128399 | secreted protein, putative | 36 | NR IR |
|  | 113785 | salivary lipid interacting protein, putative | 20 |  |
| 18 | 43508 | vitellogenin-1 | 97 | NR IR |
|  | 31546 | apolipophorin, putative | 89 |  |
|  | 43509 | vitellogenin-1 | 182 |  |
| 19 | 106321 | serine proteinase inhibitor serpin-3 | 43 | IR IS |
|  | 18420 | Actin | 42 |  |
|  | 106322 | serine proteinase inhibitor serpin-3 | 43 |  |
|  | 122308 | Longipain | 33 |  |
|  | 39114 | cathepsin L-like cysteine proteinase B | 38 |  |
| 20 | 106321 | serine proteinase inhibitor serpin-3 | 43 | IR |
|  | 18420 | Actin | 42 |  |
|  | 106322 | serine proteinase inhibitor serpin-3 | 43 |  |
|  | 122308 | Longipain | 33 |  |
|  | 39114 | cathepsin L-like cysteine proteinase B | 38 |  |
| 21 | 39114 | cathepsin L-like cysteine proteinase B | 38 | IR |
|  | 122308 | Longipain | 33 |  |
|  | 106322 | serine proteinase inhibitor serpin-3 | 43 |  |
|  | 18420 | Actin | 42 |  |
| 22 | 22310 | beta tubulin | 35 | NS IR |
|  | 7197 | beta tubulin | 53 |  |
|  | 18420 | Actin | 42 |  |
|  | 4388 | F0F1-type ATP synthase, beta subunit | 59 |  |
|  | 22306 | beta tubulin | 50 |  |
| 24 | 111630 | glutathione S-transferase | 31 | NR IR |
|  | 67998 | ENSANGP00000022132 | 25 |  |
|  | 83247 | putative salivary secreted protein | 25 |  |
|  | 8103 | secreted salivary gland peptide, putative | 25 |  |
|  | 26121 | selenium dependent salivary glutathione peroxidase | 18 |  |
| 25 | 8103 | secreted salivary gland peptide, putative | 25 | NR |
|  | 111630 | glutathione S-transferase | 31 |  |
| 26 | 8103 | secreted salivary gland peptide, putative | 25 | IS IR |
| 27 | 8103 | secreted salivary gland peptide, putative | 25 | NR IR NS |
| 28 | 13343 | translation elongation factor EF-1 alpha-Tu | 51 | NS IS |
| 29 | 13343 | translation elongation factor EF-1 alpha-Tu | 51 | NS IS NR IR |
|  | 14491 | cathepsin D2 | 42 |  |
| 30 | 106322 | serine proteinase inhibitor serpin-3 | 43 | IR |
|  | 174664 | putative secreted protein | 21 |  |
|  | 25106 | Enolase | 43 |  |
| 31 | 178127 | putative secreted protein | 21 | NR IR |
|  | 164102 | secreted protein | 23 |  |
|  | 32190 | uncharecterized protein | 17 |  |
|  | 164103 | secreted protein | 23 |  |
|  | 174664 | putative secreted protein | 21 |  |
| 32 | 171393 | putative secreted protein | 18 | NR IR |
|  | 174664 | putative secreted protein | 21 |  |
| 33 | 178127 | putative secreted protein | 21 | NR IR |
|  | 164103 | secreted protein | 23 |  |
|  | 20757 | 14-3-3 CG17870-PA, isoform A isoform 2 | 27 |  |
|  | 171393 | putative secreted protein | 18 |  |
|  | 174664 | putative secreted protein | 21 |  |
| 34 | 174664 | putative secreted protein | 21 | NR IR |
|  | 178127 | putative secreted protein | 21 |  |
|  | 20757 | 14-3-3 CG17870-PA, isoform A isoform 2 | 27 |  |
|  | 171393 | putative secreted protein | 18 |  |
| 35 | 178127 | putative secreted protein | 21 | IR |
|  | 171393 | putative secreted protein | 18 |  |
|  | 108605 | putative secreted protein | 24 |  |
| 36 | 113785 | salivary lipid interacting protein, putative | 20 | NR IR |
| 37 | 4142 | heavy-chain filboin, putative | 44 | NR IR |
|  | 31546 | apolipophorin, putative | 89 |  |
|  | 43507 | vitellogenin-1 | 158 |  |
| 38 | 121267 | hypothetical protein BRAFLDRAFT_287019 | 45 | NS |
|  | 21956 | heat shock protein, putative | 71 |  |
| 40 | 9166 | Protein disulfite isomerase-2 | 39 | IR |
|  | 30215 | chaperonin subunit, putative | 65 |  |
|  | 10534 | protein disulfite isomerase-2 | 39 |  |
| 42 | 184392 | putative secreted protein | 20 |  |
| 44 | 172281 | microplusin preprotein-like | 15 |  |
|  | 36482 | secreted protein, putative | 18 |  |
| 45 | 172281 | microplusin preprotein-like | 15 |  |
| 46 | 172281 | microplusin preprotein-like | 15 |  |
|  | 188986 | putative secreted protein | 17 |  |
|  | 125105 | neutrophil elastase inhibitor | 12 |  |
| 47 | 188986 | putative secreted protein | 17 |  |
|  | 125105 | neutrophil elastase inhibitor | 12 |  |
| 50 | 43036 | hypothetical protein AaeL_AAEL011663 | 63 |  |
| 54 | 125105 | neutrophil elastase inhibitor | 12 |  |
| 56 | 54809 | putative secreted cystatin | 15 |  |
| 57 | 75296 | alpha tubulin, putative | 51 |  |
|  | 125558 | ML domain-containing protein, putative | 12 |  |
|  | 18420 | Actin | 42 |  |
| 59 | 34860 | fatty acid-binding protein FABP, putative | 15 |  |
|  | 117512 | neutrophil elastase inhibitor | 16 |  |
| 60 | 117512 | neutrophil elastase inhibitor | 16 |  |
| 62 | 77570 | putative salivary secreted protein | 25 |  |
| 63 | 85307 | putative salivary secreted protein | 27 |  |
| 64 | 174664 | putative secreted protein | 21 |  |
|  | 178117 | putative secreted protein | 20 |  |
| 65 | 178117 | putative secreted protein | 20 |  |
| 66 | 178117 | putative secreted protein | 20 |  |
| 67 | 83247 | putative secreted protein | 25 |  |
| 72 | 106321 | serine proteinase inhibitor serpin-3 | 43 |  |
|  | 6955 | serpin-2 precursor | 36 |  |
| 73 | 106321 | serine proteinase inhibitor serpin-3 | 43 |  |
| 74 | 106321 | serine proteinase inhibitor serpin-3 | 43 |  |
|  | 6955 | serpin-2 precursor | 36 |  |
|  | 106322 | serine proteinase inhibitor serpin-3 | 43 |  |
|  | 39114 | cathepsin L-like cysteine proteinase B | 38 |  |
|  | 18420 | Actin | 42 |  |
|  | 10098 | conserved hypothetical protein | 39 |  |
| 75 | 106321 | serine proteinase inhibitor serpin-3 | 43 |  |
|  | 6955 | serpin-2 precursor | 36 |  |
| 76 | 77570 | putative salivary secreted protein | 25 |  |
| 77 | 43509 | vitellogenin-1 | 182 |  |
| 78 | 149048 | neutrophil elastase inhibitor | 12 |  |
|  | 125105 | neutrophil elastase inhibitor | 12 |  |
| 79 | 75296 | alpha tubulin, putative | 51 |  |
|  | 22306 | beta tubulin | 50 |  |
|  | 149048 | neutrophil elastase inhibitor | 12 |  |
|  | 125105 | neutrophil elastase inhibitor | 12 |  |
| 80 | 149048 | neutrophil elastase inhibitor | 12 |  |
|  | 125105 | neutrophil elastase inhibitor | 12 |  |
| 81 | 149048 | neutrophil elastase inhibitor | 12 |  |
|  | 125105 | neutrophil elastase inhibitor | 12 |  |
|  | 92814 | asparaginyl-tRNA synthetase | 59 |  |
| 82 | 149048 | neutrophil elastase inhibitor | 12 |  |
|  | 125105 | neutrophil elastase inhibitor | 12 |  |
| 85 | 133320 | unnamed protein product | 6 |  |
|  | 6026 | ATP-dependent RNA helicase, putative | 99 |  |
|  | 107453 | guanylate-binding protein, putative | 65 |  |
|  | 8831 | putative cement protein | 27 |  |
|  | 117585 | Ras-associated protein 2-like CG3204-PA, isoform A | 21 |  |
|  | 125369 | hypothetical protein IscW_ISCW023265 | 37 |  |
|  | 57845 | coatomer subunit delta | 58 |  |
|  | 80968 | ADP-ribosylation factor interacting protein, putative | 27 |  |
| 87 | 106981 | cyclophilin B precursor, putative | 21 |  |
| 88 | 172281 | microplusin preprotein-like | 15 |  |
| 89 | 125105 | neutrophil elastase inhibitor | 12 |  |
| 90 | 38904 | secreted protein, putative | 38 |  |
|  | 8303 | matricellular protein osteonectin-SPARC-BM-40, putative | 35 |  |
|  | 128399 | secreted protein, putative | 36 |  |
| 91 | 128399 | secreted protein, putative | 36 |  |
| 92 | 61864 | putative salivary secreted protein | 25 |  |
|  | 128399 | secreted protein, putative | 36 |  |
|  | 77570 | putative salivary secreted protein | 25 |  |
|  | 54777 | putative thyropin precursor | 23 |  |
| 93 | 178127 | putative secreted protein | 21 |  |
| 94 | 174677 | putative secreted protein | 19 |  |
|  | 178117 | putative secreted protein | 20 |  |
|  | 203249 | putative secreted protein | 11 |  |
|  | 178127 | putative secreted protein | 21 |  |
|  | 176335 | putative secreted protein | 14 |  |
| 95 | 21956 | heat shock protein, putative | 71 |  |
| 96 | 171727 | lysosomal acid phosphatase, putative | 26 |  |
|  | 113489 | lospin 8 | 43 |  |
| 97 | 122308 | Longipain | 33 |  |
|  | 39114 | cathepsin L-like cysteine proteinase B | 38 |  |
|  | 106322 | serine proteinase inhibitor serpin-3 | 43 |  |
|  | 18420 | Actin | 42 |  |
|  | 6955 | serpin-2 precursor | 36 |  |
|  | 106321 | serine proteinase inhibitor serpin-3 | 43 |  |
| 98 | 6064 | hypothetical protein IscW_ISCW004528 | 73 |  |
|  | 106321 | serine proteinase inhibitor serpin-3 | 43 |  |
|  | 106322 | serine proteinase inhibitor serpin-3 | 43 |  |
|  | 43509 | vitellogenin-1 | 182 |  |
| 99 | 43509 | vitellogenin-1 | 182 |  |
| 100 | 105653 | esterase, putative | 60 |  |
| 101 | 112943 | cathepsin C precursor | 51 |  |
|  | 105653 | esterase, putative | 60 |  |

Salivary proteins were obtained from saliva of female *R. microplus* ticks feeding on genetically susceptible hosts. Sera employed were obtained from naïve and/or two-times infested genetically tick-susceptible and/or -resistant bovines. Criteria employed to identify each sequence: minimum of 1 peptide presenting with 90% probability of being the protein and 90% probability of being the peptide. 1) CDS: coding sequences generated by the sialotranscriptome of *R. microplus* described and deposited at GenBank - NCBI (BioProject ID PRJNA329522). 2) Molecular weight furnished by Scaffold software. 3) Abbreviations for sources of sera and samples: NR: pooled sera collected from four tick-naïve, genetically resistant Nelore; IR: pooled sera from four genetically resistant, twice-infested Nelore bovines at the end of the infestation; NS: pooled sera collected from four tick-naïve, genetically susceptible Holstein bovines; IS: pooled sera from four genetically susceptible, twice-infested Holstein bovines at the end of the infestation. The table is formatted according spot number described at Figure 5. In some instances there are more than one protein identified by spot collected.
